# An epidemiological and clinicopathological study of type 1 vs. type 2 morphological subtypes of papillary renal cell carcinoma– results from a nation-wide study covering 50 years in Iceland

**DOI:** 10.1186/s12894-024-01494-9

**Published:** 2024-05-13

**Authors:** Thorri Geir Runarsson, Andreas Bergmann, Gigja Erlingsdottir, Vigdis Petursdottir, Leon Arnar Heitmann, Aevar Johannesson, Viktor Asbjornsson, Tomas Axelsson, Rafn Hilmarsson, Tomas Gudbjartsson

**Affiliations:** 1 Faculty of Medicine, University of Iceland, Reykjavik, Iceland; 2 Department of Urology and Surgery in Landspitali University Hospital, Reykjavik, Iceland; 3 Department of Urology in Danderyd Hospital, Stockholm, Sweden; 4 Department of Pathology in Landspitali University Hospital, Reykjavik, Iceland; 5 Department of Statistics in University of Iceland, Reykjavik, Iceland; 6 Department of Surgery and Urology, Landspitali University Hospital, University of Iceland, Hringbraut IS-101, Reykjavik, Iceland

**Keywords:** Papillary renal cell carcinoma, Renal cell carcinoma, Histology, Subtyping, Kidney cancer, Renal cancer, Incidence, Prognosis, Survival, Mortality

## Abstract

**Introduction:**

Papillary renal cell carcinoma (pRCC) is the second most common histology of renal cell carcinoma (RCC), accounting for 10–15% of cases. Traditionally, pRCC is divided into type 1 and type 2, although this division is currently debated as a prognostic factor of survival. Our aim was to investigate the epidemiology and survival of the pRCC subtypes in a whole nation cohort of patients during a 50-year period.

**Materials and methods:**

A Population based retrospective study including consecutive cases of RCC in Iceland from 1971–2020. Comparisons were made between histological classifications of RCC, with emphasis on pRCC subtypes (type 1 vs. 2) for outcome estimation. Changes in RCC incidence were analyzed in 5-year intervals after age standardization. The Kaplan–Meier method and Cox regression were used for outcome analysis.

**Results:**

A total of 1.725 cases were identified, with 74.4%, 2.1% and 9.2% having clear cell (ccRCC), chromophobe (chRCC), and pRCC, respectively. The age standardized incidence (ASI) of pRCC was 1.97/100.000 for males and 0.5/100.000 for females, and the proportion of pRCC increased from 3.7% to 11.5% between the first and last intervals of the study (*p* < 0.001). Age standardized cancer specific mortality (ASCSM) of pRCC was 0.6/100.000 and 0.19/100.000 for males and females, respectively. The annual average increase in ASI was 3.6% for type 1 pRCC, but the ASI for type 2 pRCC and ASCSM for both subtypes did not change significantly. Male to female ratio was 4.4 for type 1 pRCC and 2.3 for type 2. The average tumor size for type 1 and 2 was 58.8 and 73.7 mm, respectively. Metastasis at diagnosis was found in 8.7% in the type 1 pRCC, compared to 30.0% of patients with type 2 pRCC (*p* < 0.001). Estimated 5-year cancer-specific survival (CSS) were 94.4%, 80.7%, and 69.3% for chRCC, pRCC and ccRCC, respectively (*p* < 0.001). For the pRCC subtypes, type 1 was associated with better 5-year CSS than type 2 (86.3% vs. 66.0%, *p* < 0.001), although this difference was not significant after adjusting for cancer stage and grading.

**Conclusions:**

pRCC histology was slightly less common in Iceland than in other countries. Males are more than three times more likely to be diagnosed with pRCC, compared to other RCC histologies. The subtype of pRCC was not found to be an independent risk factor for worse survival, and as suggested by the most recent WHO Classification of Urinary Tumors, grade and TNM-stage seem to be the most important factors for estimation of survival for pRCC patients.

**Supplementary Information:**

The online version contains supplementary material available at 10.1186/s12894-024-01494-9.

## Introduction

Renal cell carcinoma (RCC) accounts for 85% of malignant kidney tumors. Clear cell RCC (ccRCC) is the most common histological subtype (75–80%), followed by papillary RCC (pRCC) in 10–15% of cases, but other subtypes, such as chromophobe RCC (chRCC) and collecting duct carcinoma, are less common [1–3]. Traditionally, pRCC is divided into type 1 and type 2 subtypes based on histological differences, where type 1 pRCC is often more multifocal with small cells arranged in papillary and tubular structure with basophilic cytoplasm and an oval nuclei, while type 2 pRCC is more heterogenous with large eosinophilic cells in a papillary structure and large spherical nuclei [4].

In spite of their histological distinctions, differences in clinical outcome of RCC subtypes have been debated, with the exception of the more benign chRCC [5–7]. In a nation-wide study from Iceland, histology was not found to be an independent prognostic factor after adjusting for nuclear grade and TNM stage, although pRCCs were not analyzed based on conventional subtyping [8]. Furthermore, numerous other studies have not found the subtype of pRCC to be an independent predictor of survival, that is after TNM-stage and nuclear grade have been taken into account [9–15]. As type 2 pRCCs are often more advanced at diagnosis and tend to have a more aggressive clinical course than type 1 pRCCs, these findings are conflicting [4, 16]. On the other hand, recent molecular studies suggest that type 2 pRCC is not a single, well-defined entity, but rather three individual subgroups with different molecular and genetic profiles. Common alterations in type 2 pRCC are found in the *CDKN2A*, *SETD2*, *TFE3*, and *FH* genes, whereas type 1 pRCC predominantly includes *MET* alterations and the others are typically not present [17]. Recently, these genetic and molecular subclassification of type 2 pRCC have been implemented in the 5th Edition of WHO classification of *Urinary and Male Genital Tumors*, with less weight on the conventional type 1 and 2 morphological subtyping [18]. Regardless, the histological subtyping into pRCC types 1 and 2 is used in many centers worldwide, including in Iceland.

To date, most studies comparing pRCC subtypes are derived from single centers and almost exclusively include patients that have been operated on [9, 11–16, 19]. Furthermore, data on the change in incidence and mortality of the different pRCC subtypes is scarce. We therefore investigated the epidemiology and clinical course of the major histological RCC classifications over a 50-year period in Iceland, with emphasis on the incidence and clinical outcome of conventional pRCC subtypes.

## Materials and methods

### Study design

A retrospective population-based study including consecutive patients diagnosed with RCC between January 1st, 1971, and December 31st, 2020, at Landspitali University Hospital in Iceland – the sole tertiary institution performing nephrectomies in Iceland.

### Data collection

Patients were identified in three different databases. An internal operational and diagnosis registry at Landspitali University Hospital was searched for ICD codes for RCC; using ICD-C64 and D41.0 if the tumor was of unknown origin. To ascertain that all cases of RCC were included, the search results were compared to the database of The Icelandic Cancer Registry, a centralized government operated institution that documents all cancer diagnoses in Iceland.

Patient data were derived from hospital charts and pathological reports at Landspitali. Data on age, gender and symptoms at diagnosis were obtained from patient charts. Both localized symptoms, such as hematuria, flank pain and abdominal mass, as well as systemic symptoms, such as fever, weight loss, night sweats and pain from metastatic lesions, were collected. Imaging studies that led to the diagnosis were listed, as well as the type of surgery performed. Metastasis work up was mostly done by computed tomography (CT) of the chest, abdomen, and brain, depending on symptoms. Patients were staged and graded according to the 2017, 8th TNM-staging system and the Fuhrman grading system [20–22].

The primary endpoint was cancer specific survival (CSS), and secondary endpoints were the incidence and overall survival (OS) of type 1 and type 2 pRCCs. All cases were followed up for OS and CSS, which was based on the diagnosis date until the day of death, or 14th March 2023, whichever came first. The diagnosis date was defined by the date of the pathological report. The Icelandic Cause of Death Registry provided data on cause of death, which was classified as either RCC related or not.

### Histological evaluation

All patients with pRCC were categorized into type 1 and type 2 by a senior pathologist, using the WHO definition from 2016 [23]. If the diagnosis was ambiguous or the pathology showed a hybrid or mixed pattern, two or three other senior pathologists reviewed the tissues samples. If no pathological pattern was dominant, the cases were not subdivided and instead classified as hybrid or mixed pattern. Patients diagnosed with type 1 pRCC and those diagnosed with type 2 pRCC were compared to each other but also the two other major histology groups (ccRCC and chRCC). To investigate trends, the 50-year study period was divided into ten 5-year time periods.

#### Statistical analysis

Data were collected with Microsoft Excel and statistical analysis were performed using R version 4.2.2 (Wien, Austria), and R Studio (version 4.2.2). Age standardized incidence (ASI) was calculated based on Icelandic population data from the Icelandic Bureau of Statistics with 2020 as a reference year. During the study period, the population ranged from 204.934 in January 1st 1971 to 368.792 in December 31st 2020 with an average of 274.216 [24].

Poisson regression was used to estimate trends in incidence and mortality. Both OS and CSS were estimated with the Kaplan–Meier method and compared using a stratified log-rank test between groups. Prognostic factors for survival were evaluated using multivariable Cox regression and presented with hazard ratios (HR) and 95% confidence intervals (CI) for each covariate, adjusting for potential confounding variables. The covariates included in our analysis were age, gender, TNM stage, Fuhrman grade, period of diagnosis, pRCC subtypes, and the type of clinical diagnosis (incidental vs. symptomatic). The Chi-square was used for comparison of categorical variables, and the Fischer ‘s exact test was used if the expected value was less than 5. Analysis of variance was used for comparison of numerical variables.

#### Ethics statement

The study was approved by the National Bioethics Committee of Iceland. As individual patients were not identified, the need for individual informed consent was waived by the same ethics Committee.

## Results

During the 50-year study period, 1.725 patients were diagnosed with RCC, including 1.284 (74.4%) patients with histologically proven ccRCC, 158 (9.2%) patients with pRCC and 37 patients with chRCC (2.1%). The median follow up time was 59 months (4.9 years), with a mean of 94 and spanning 1 to 466 months. For incidence and mortality calculations, all 158 pRCC patients were included. In our subtype comparison of pRCC, 8 hybrid/mixed type 1 and type 2 pRCC cases were excluded and 7 pRCC patients where subtyping was missing were also excluded, leaving 143 pRCCs for subtype comparison. Furthermore, RCCs diagnosed incidentally at autopsy were excluded.

Out of 143 pRCC cases included, 103 were type 1 (72.0%) and 40 type 2 (28.0%). The incidence of pRCC increased from 6.1% in 1971–1980 to 13.1% in 2011–2020 (*p* < 0.001).

**Fig. 1:**
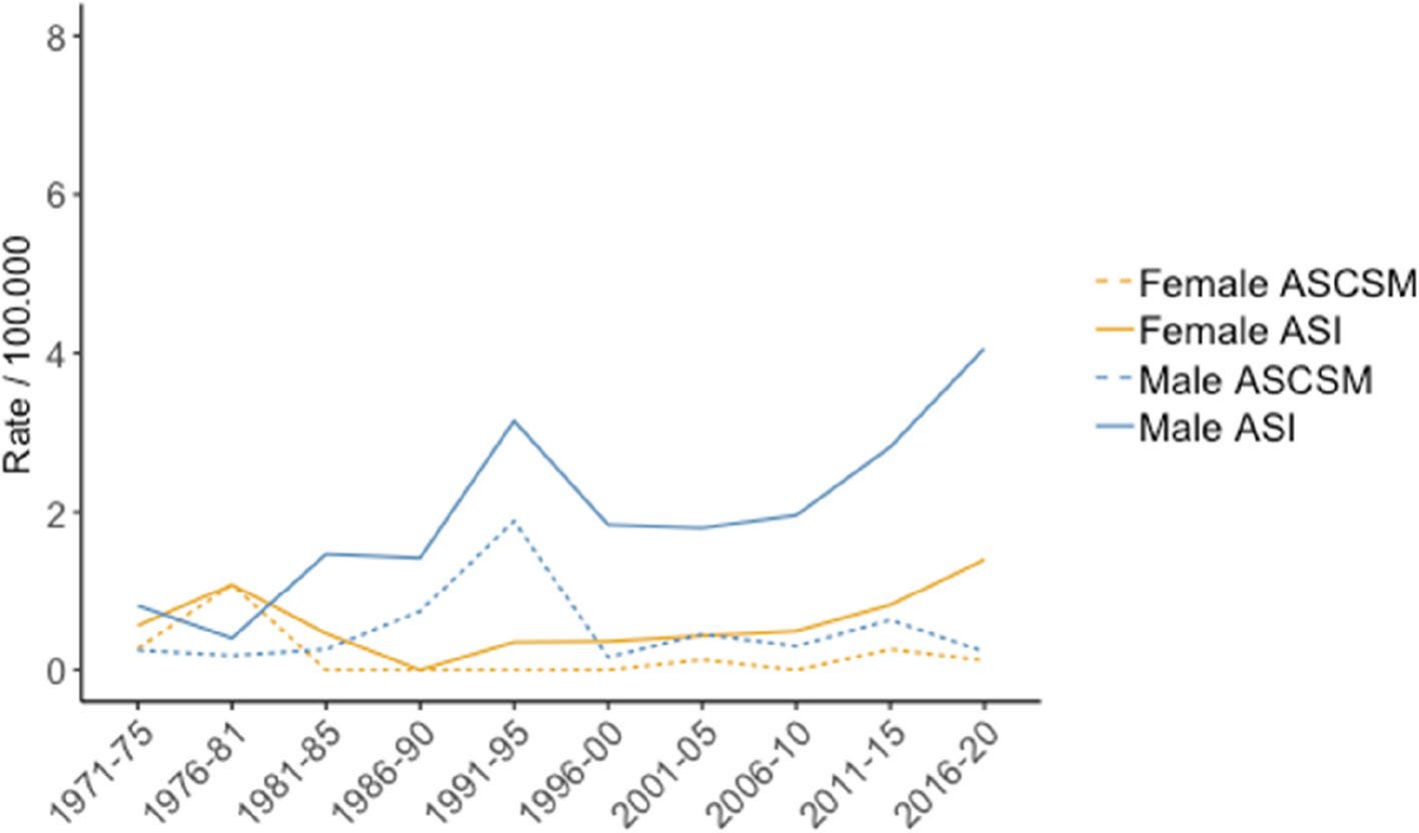
Age standardized incidence (ASI) and age standardized cancer specific mortality (ASCSM) for papillary RCC for males and females per 100.000 inhabitants during 1971–2020. Type 1 and 2 pRCC are analyzed together

During the 50-year study period, the ASI was 1.28/100.000 for the whole group, 1.97/100.000 for males and 0.60/100.000 for females, respectively (Fig. 1). During the last 5-year period (2016–2020) the ASI was 4.06 and 1.4 for males and females, respectively. For the whole study period the average annual increase in ASI was 2.8% (*p* = 0.002); 3.4% (*p* < 0.001) for males and 2.5% (*p* = 0.053) for females. The overall ASCSM was 0.34/100.000, 0.5/100.000 and 0.19/100.000 for males and females, respectively, and did not change significantly during the 50-year study period.

**Fig. 2:**
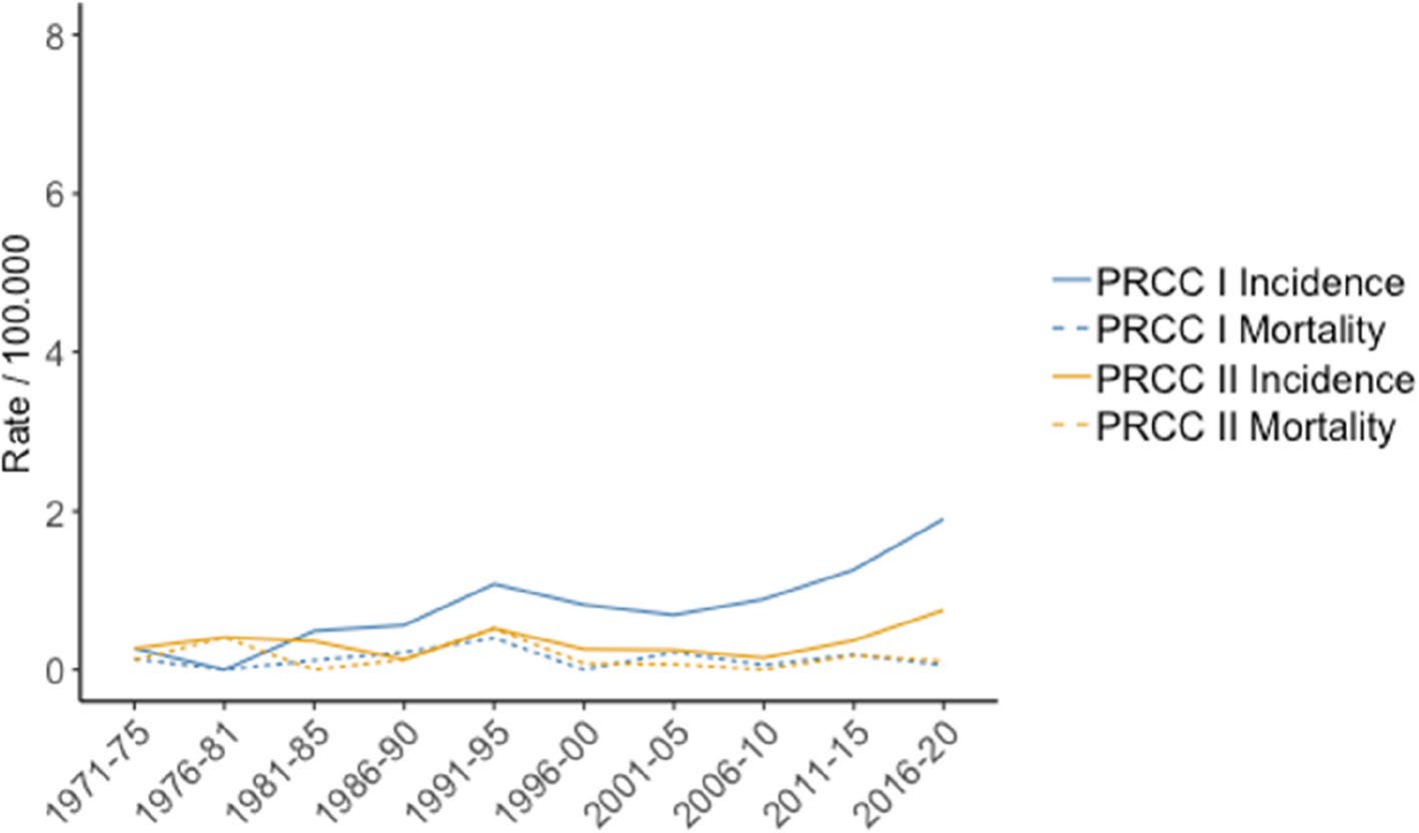
Age standardized incidence (ASI) and cancer specific mortality (ASCSM) for papillary 1 (pRCC-1) and papillary 2 RCC (pRCC-2) per 100.000 inhabitants during 1971–2020

ASI and ASCSM for type 1 pRCC and type 2 pRCC are shown in Fig. 2. The overall ASI were 0.8 and 0.35 for type 1 and type 2 pRCC, while the ASCSM were 0.14 and 0.16 per 100.000, respectively. For type 1 pRCC, the average annual increase in ASI was 3.6% (*p* = 0.001) for the whole group, or 5.1% (*p* < 0.001) and 4.0% (*p* = 0.027) for males and females, respectively. No significant changes in ASI were found for type 2 pRCCs although a trend for increase in males (2.5% *p* = 0.088) was observed. Finally, the ASCSM for both type 1 and 2 pRCC did not change significantly during the study period.

### Comparison of type 1 vs. 2 pRCCs with ccRCC and chRCC

Comparison of type 1 and 2 pRCC is shown in Table 1, along with comparison with the two other major histological subtypes. Compared to females, males were significantly more likely to have pRCC, with a male to female ratio of 4.4, 2.3, 1.6, and 1.3 for type 1 pRCC, type 2 pRCC, ccRCC and chRCC, respectively. Most patients were diagnosed during the last decade of the study period, or 50.5% of the type 1 pRCCs, 47.5% of type 2 pRCCs 2, 30.6% of the ccRCCs and 40.5% of the chRCCs (*p* < 0.001). The average size of type 1 pRCC tumors was 58.8 mm compared to 73.7 mm for type 2 pRCC (*p* = 0.06), with the sizes of ccRCC and chRCCs being in between. Furthermore, the average size for type 1 and 2 pRCCs were 49.9 mm and 55.6 mm in 2011–2020, compared to 80.0 mm and 91.5 mm in 1971–1980, respectively.

**Table 1:** Patient demographics for patients with type 1 and 2 pRCC, ccRCC and chRCC. Data is presented as numbers with percentages in parenthesis unless otherwise stated. *P*-value refers to comparison of the four histology types

Variable	Papillary 1 (PRCC-1) *N* = 103	Papillary 2 (PRCC-2) *N* = 40	Clear cell (ccRCC) *N* = 1284	Chromophobe (chRCC) *N* = 37	*p*-value
**Mean age (years)**	65.4 ± 10.6	64.8 ± 13.7	64.3 ± 11.6	58.2 ± 15.0	**0.013**
**Males (M/F ratio)**	84 (4.4)	28 (2.3)	785 (1.6)	21 (1.3)	**< 0.001**
**Laterality (Right tumors)**	44 (43)	18 (45)	644 (50)	21 (57)	0.36
**Bilateral**	2 (2)	0	22 (1.7)	0	0.92
**Tumor size (mm)**	58.8	73.7	65.5	71.4	0.12
**Operation**					
Nephrectomy	71 (69)	26 (65)	988 (77)	28 (76)	0.11
Partial nephrectomy	24 (23)	6 (15)	141 (11)	7 (19)	**0.001**
**Period**					
1971–1980	2 (2)	4 (10)	120 (9)	4 (11)	**0.03**
1981–1990	9 (9)	4 (10)	183 (14)	2 (5)	0.20
1991–2000	20 (19)	8 (20)	234 (18)	7 (19)	0.98
2001–2010	20 (19)	5 (13)	354 (28)	9 (24)	**< 0.001**
2011–2020	52 (50)	19 (48)	393 (31)	15 (41)	**< 0.001**
**TNM stage**					
1	60 (58)	16 (40)	533 (42)	16 (43)	**0.01**
2	22 (21)	5 (13)	128 (10)	10 (27)	**< 0.001**
3	12 (12)	6 (15)	315 (25)	10 (27)	**0.01**
4	9 (9)	13 (33)	308 (24)	1 (3)	**< 0.001**
**Fuhrman grade**					
1	10 (10)	1 (3)	76 (6)		0.15
2	58 (56)	16 (40)	643 (50)		0.36
3	23 (22)	16 (40)	398 (31)		0.16
**Metastasis**					
Lungs	5 (5)	5 (13)	170 (13)	1 (3)	**0.01**
Liver	1 (1)	1 (3)	58 (5)	0	0.23
Bone	3 (3)	3 (8)	120 (9)	1 (3)	0.07
Skin	0	1 (3)	22 (2)	1 (3)	0.35
Brain	0	0	20 (2)	1 (3)	0.43
**Diagnosis**					
Incidental	52 (50)	14 (35)	506 (39)	12 (32)	0.10
Symptomatic	51 (50)	26 (65)	778 (61)	25 (68)	0.10
**OS% / CSS%**					
1-year	93/93	85/85	80/82	85/97	**< 0.001/ < 0.001**^**a**^
3-year	81/89	63/69	69/75	92/94	
5-year	73/86	56/66	60/69	92/94	
10-year	61/86	40/57	45/62	85/94	

*RCC* Renal cell carcinoma*, CSS* Cancer specific survival, *OS* Overall survival

^a^Overall survival compared between groups as well as cancer specific survival

Incidentally diagnosed patients were 39.8% in total; divided into 50.5%, 35.0%, 39.4% and 32.4% for type 1 pRCC, type 2 pRCC, ccRCC and chRCC, respectively. The type 2 pRCC tumors were diagnosed at a significantly higher TNM stage and Fuhrman grade than type 1 tumors, with 58.3% of type 1 pRCC diagnosed on stage I compared to 40.0% for type 2 pRCCs. When comparing the TNM stages of type 1 and 2 pRCC patients, stage IV disease was more common in type 2 pRCC patients (*p* < 0.001).

Metastases were present in 22.1% of the RCC-patients at diagnosis, with type 1 pRCC (30.0%) being the most likely to have metastasis at diagnosis, followed by ccRCC (23.4%), pRCC-1 (8.7%) and chRCC (2.7%) (*p* < 0.001). Lung metastases were most common for all the histology groups, followed by bone and liver metastasis.

Radical nephrectomy was performed in 97 (67.8%) of the 143 pRCC patients, and partial nephrectomy in 30 (21.0%). Sixteen patients were treated conservatively, most often due to not being deemed fit for surgery. The resection rate of type 1 and 2 pRCC patients operated on was 92.2% and 80.0%, compared to 94.6% and 87.9% for chRCC and ccRCC, respectively.

### Survival and comparison of groups

**Fig. 3:**
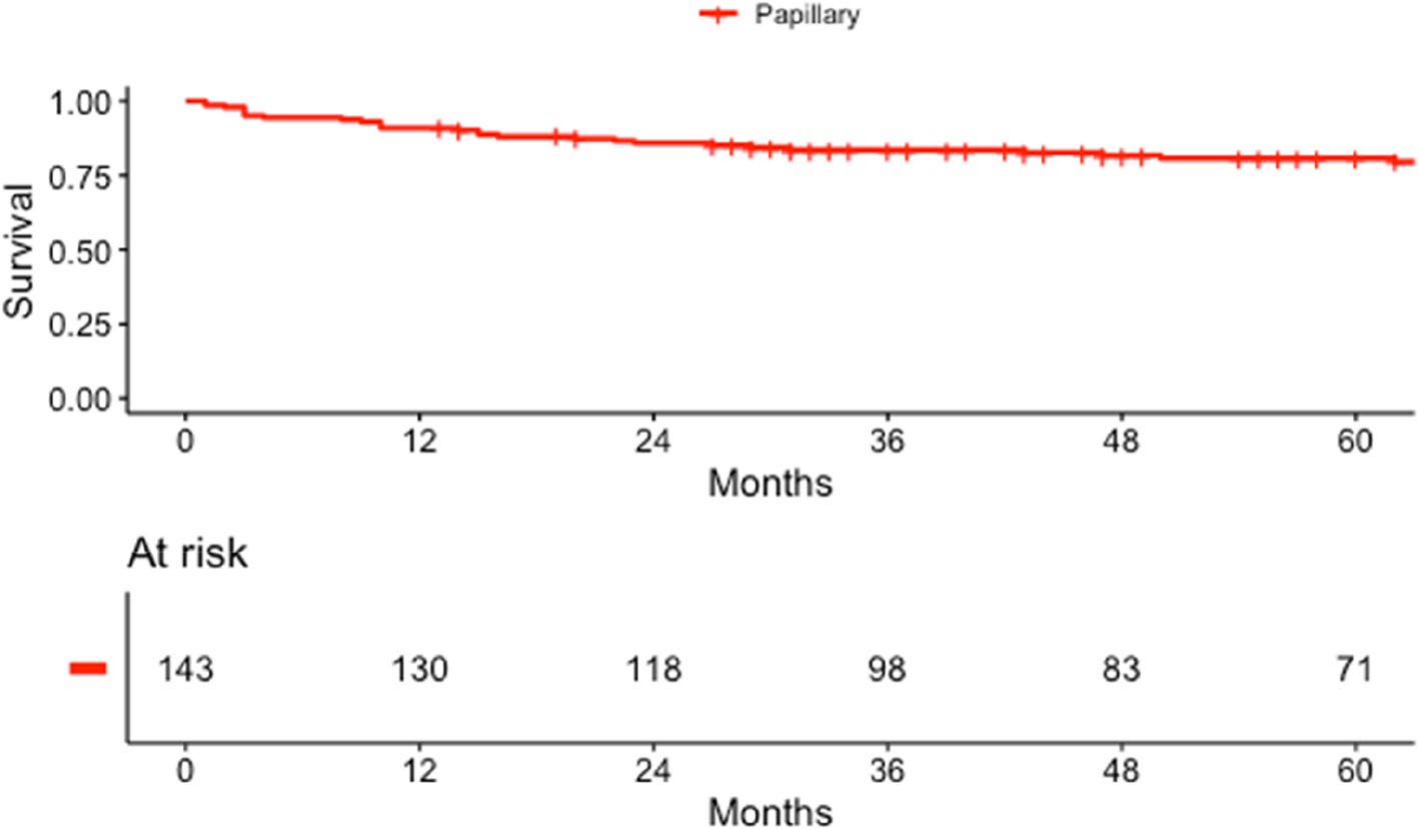
Estimated cancer specific survival (CSS) for patients with papillary RCC (type 1 and 2 analyzed together)

Estimated CSS for pRCC is shown in Fig. 3. The respective 1-year, 3-year, and 5-year CSS were 90.9% (CI: 0.86, 0.96), 83.6% (CI: 0.78, 0.9) and 80.7% (CI: 0.74, 0.88), respectively.

**Fig. 4:**
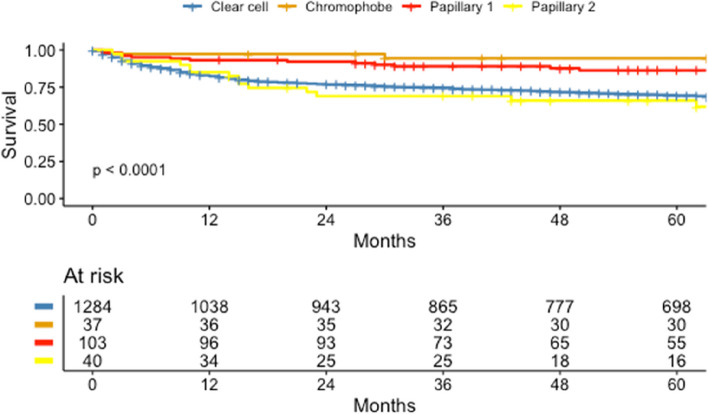
Estimated CSS for the four most common RCC histology types from 1971–2020

Figure 4 shows the difference in CSS between histology types. When pRCC subtypes were combined, they were associated with poorer CSS than chRCC (log-rank, *p* = 0.027), but higher CSS compared to ccRCC (log-rank, *p* = 0.003). Furthermore, type 1 pRCCs had better CSS than ccRCC (log-rank test, *p* < 0.001), and type 2 pRCCs, similar to ccRCC (log-rank, *p* = 0.5), but poorer than chRCC (log-rank, *p* < 0.001).

**Fig. 5:**
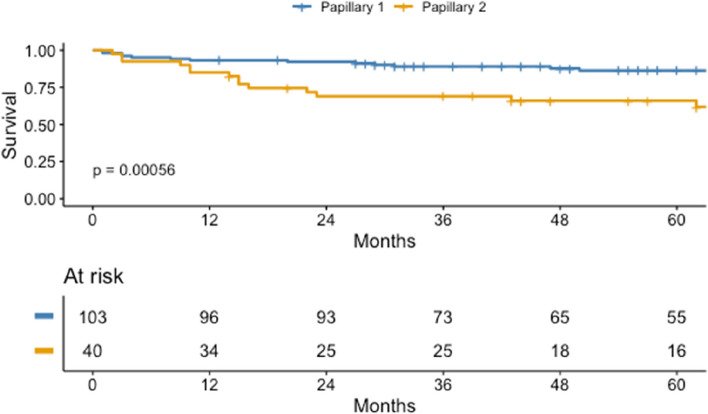
Estimated CSS for pRCC type 1 and type 2 in 1971–2020

Patients with type 1 pRCC had significantly better OS and CSS compared to type 2 pRCC patients (Fig. 5), with 1-year and 5-year CSS being 93.2% (95% CI: 0.89–0.98) and 86.3% (95% CI: 0.80–0.94) for type 1, compared to 85.0% (95% CI: 0.75–0.97) and 66.0% (95% CI: 0.53–0.83) for type 2 pRCC, respecitvely.

**Fig. 6:**
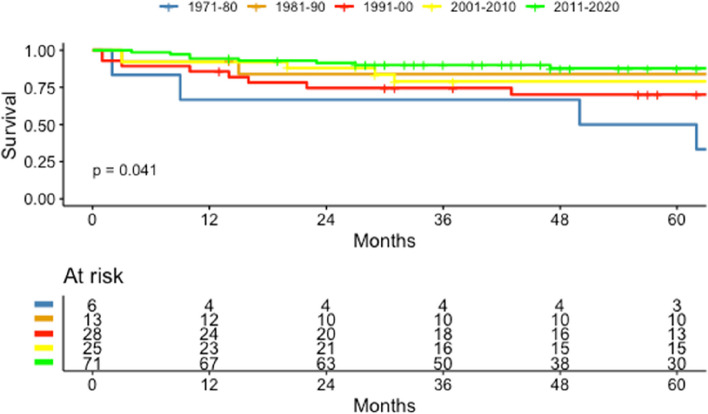
Estimated CSS for pRCC for each decade between 1971–2020

Survival was positively associated with increasing calendar years. The 1-year and 5-year CSS for pRCC was 66.7% (CI: 0.38–1.0) and 50.0% (CI: 0.23–1.0) in 1971–1980 and increased to 94.4% (CI: 0.89–1.0) and 87.8% (CI: 0.80–0.96) in 2011–2020, respectively, (Fig. 6). For stage IV disease, 5-year CSS was 2.29% (CI: 0.003–0.16) in 1971–1980 and increased to 22.4% in 2011–2020 (CI: 0.14, 0.36). Survival for the different pRCC TNM stages and Fuhrman grades is shown on Figure S1 and S2 in supplements.

### Predictors of survival

Table 2 shows a multivariable analysis of prognostic factors for CSS and OS for the pRCCs. Advanced age was independently associated with inferior survival (HR: 1.08 CSS and 1.07 OS, *p* < 0.001) as were all individual TNM stages, with stage IV disease having the lowest survival (HR: 2.44 for CSS and 10 for OS). Fuhrman grade 4 was significantly associated with inferior CSS, OS and time period; with patients treated 2011–2020 having more favorable CSS (HR: 0.13 for CSS) but not OS; compared to the first period (1971–1980). Incidental diagnosis did not predict survival and neither did pRCC subtypes (*p* = 0.78 for CSS and 0.22 for OS).

**Table 2:** Cox multivariable analysis showing prognostic factors for survival and hazard ratios of overall deaths and cancer-specific deaths along with 95% confidence intervals and significance of each variable

		**Overall deaths**		**Deaths from RCC**^**a**^	
**Variable**	**N**	**Hazard ratio (95% CI**^**b**^**)**	***p*-value**	**Hazard ratio (95% CI)**	***p*-value**
**Age**	129	1.07 (1.04, 1.10)	**< 0.001**	1.08 (1.03, 1.13)	**0.002**
**Gender**					
Males	101	Reference		Reference	
Females	28	1.03 (0.57, 1.88)	0.92	0.92 (0.33, 2.56)	0.87
**TNM**					
I	66	Reference		Reference	
II	26	2.12 (1.05, 4.30)	**0.036**	22.38 (2.54, 197.2)	**0.005**
III	16	3.46 (1.46, 8.21)	**0.005**	23.12 (2.04, 262.15)	**0.011**
IV	21	10.25 (4.16, 25.23)	**< 0.001**	244.33 (21.03, 2838.28)	**< 0.001**
**Fuhrman grade**					
1	11	Reference		Reference	
2	74	1.55 (0.37, 6.55)	0.551	1.81 (0.13, 25.52)	0.662
3	39	1.04 (0.24, 4.59)	0.959	1.28 (0.11, 15.05)	0.847
4	5	13.09 (2.33, 73.37)	**0.003**	22.99 (1.66, 318.8)	**0.019**
**Period**					
1971–80	6	Reference		Reference	
1981–90	13	0.71 (0.19, 2.60)	0.608	0.40 (0.06, 2.91)	0.366
1991–00	28	1.65 (0.52, 5.28)	0.395	0.70 (0.15- 3.38)	0.655
2001–10	24	0.87 (0.26, 2.95)	0.822	0.41 (0.07, 2.37)	0.319
2011–20	58	0.40 (0.11, 1.39)	0.149	0.13 (0.02, 0.74)	**0.022**
**Diagnosis**					
Incidental	60	Reference		Reference	
Symptomatic	69	0.69 (0.37, 1.30)	0.249	0.45 (0.12, 1.67)	0.231
**Histology**					
Papillary 1	93	Reference		Reference	
Papillary 2	36	1.49 (0.79, 2.80)	0.215	0.86 (0.30, 2.46)	0.77

^a^*RCC* Renal cell carcinoma

^b^*CI* Confidence interval

## Discussion

In this study, we investigated the outcomes and epidemiological changes of RCC diagnoses in a nationwide cohort spanning 50-years. We found that the incidence for the two morphological pRCC subtypes increased alongside an upward trend in survival, but unchanged mortality. Finally, although the survival rates of individual pRCC subtypes differed, the subtyping itself was not associated with worse CSS or OS after adjusting for nuclear grade, and TNM staging. Our results support prior findings on the impact of pRCC subtyping on survival and provide a more robust transferability due to our population detectability of cases [9–15].

### Survival and prognostic factors

PRCC had inferior survival to chRCC and more favorable survival than ccRCC, both of which has previously been reported in several studies, including from Iceland [5, 8, 25, 26]. Furthermore, type 2 pRCC patients had poorer 5-year OS and CSS than type 1 pRCC, which is also supported in previous studies [16, 27, 28]. A particularly positive finding is the positive association between CSS of pRCC and advanced calendar year. This development may have multiple explanations, including improved surgical techniques, such as minimal invasive approach replacing conventional open surgery [29]. However, the increase in the diagnosis of lower stage tumors, mainly due to an increase in incidental diagnoses, is probably a key contributor [30]. Finally, advances in the treatment for stage IV diseases with new targeted therapies may also have played a role, as reflected by the significantly improved survival of the stage IV disease [31, 32].

As in most RCC studies, the TNM stage of pRCC proved to be the strongest prognostic factor in multivariable analysis for both OS and CSS [33, 34]. This was also observed for Fuhrman grade 4, although that group only consisted of 5 patients. Several studies have reported nuclear grade as predictor for better outcome, including the more recent WHO/ISUP grading system [34–37]. However, in many of these studies, its significance in multivariable analysis diminishes after correcting for TNM stages [33].

Advanced age was an independent prognostic factor for both OS and CSS, which correlates to the findings of Ledezma et al. [11]. Importantly, when adjusting for age, gender, stage, grade, time period and type of diagnosis in the Cox multivariable analysis, the papillary subtype (type 1 or 2) did not predict either OS or CSS. Similar results have been reported previously [9–12, 15]. This was additionally supported by a meta-analysis of Yang et al., that did not find a difference in OS between pRCC subtypes, and the authors therefore concluded that grade and histology architecture should be used to predict OS rather than histological subtyping [12]. In contrast, Wong et al., with a cohort of 509 pRCC patients, found that pRCC subtyping was associated with better survival of type 1 pRCC tumors (HR: 8.2 *p* < 0.001) [16]. Importantly, this study did not include metastatic pRCC, which constituted 30.0% of type 2 pRCC and 8.7% of type 1 pRCC patients at diagnosis in the present study.

### Incidence

Papillary RCC constituted only 9.2% of RCCs in this study, which is a slightly higher ratio than in the SEER study (8.3%) (North American Surveillance, Epidemiology and End Results) by Saad et al. [38]. However, the proportion of pRCC in the SEER study, as well as the present study, is somewhat lower than reported in most epidemiologicalstudies, where it usually ranges from 10 to 15% [1, 2, 39]. The relatively low proportion of pRCC in Iceland may be explained by our nation-wide inclusion of cases. Although the SEER-study was not population based, it did include more than 100.000 patients with 8,730 pRCC cases [38]. Furthermore, pRCC has been shown to vary by race, with higher prevalence in black people, which represents a small percentage (< 1%) of the Icelandic population [40].

During the 50-year study period, the average ASI was 1.28 for the whole group; 1.97 and 0.60 per 100.000 males and females. This is in line with a study by Palumbo et al. that reported the ASI of 1.4/100.000 person years from 2001–2016 based on data from the SEER study [41]. Furthermore, they reported an average annual change in ASI of 4.9% for the whole group, 4.7% for males, and 5.4% for females during the periods 2001–2016, which is comparable to our results [41]. Another study from Saad et al. reported a steeper increase in incidence between 1992 and 2015 (9,1%), yet with a plateau from 2008 [38]. On the other hand, ASCSM in the present study did not change over the study period, which would imply that survival is improving, as ASI increased while mortality was unchanged.

The reasons for the increased ASI of pRCC is not fully understood, but similar trends have been observed for both ccRCC and chRCC. Known risk factors, such as obesity and rising age, may play a role, but a sharp rise in incidental detection due to more frequent use of abdominal imaging for unrelated disease has probably contributed to this change [42]. Another possibility is that the variable size criteria for the diagnosis of small, low grade, papillary tumors (5-15 mm), could also play a role [43, 44]. In addition, the prevalence of end stage kidney disease, which has shown a strong association to pRCC, has increased in the past decades [45, 46]. It should also be noted that the profile of risk factors has changed considerably in recent years, which limits inference. As an example, smoking has been eradicated in Iceland over the past decade, while obesity has increased to 25% of males and 27% of females, which must be regarded as high in European comparison [47, 48]. To the best of our knowledge, ASI and ASCSM have not been estimated for pRCC subtypes in a whole population, which also makes comparison with other studies more difficult.

Male sex was most common for all major histological RCC subtypes, which is in line with other studies, however, the proportion of males with type 1 pRCC was notably high [40, 49, 50]. Age, laterality, and tumor size were comparable between the pRCC subtypes. However, some studies have shown that type 1 pRCC is diagnosed at an earlier age than type 2, which influences tumor size at diagnosis [16]. Furthermore, bilateral pRCCs were only present in 2% of pRCC patients, although they have been described in up to 4% in the literature [51].

Patients with type 2 pRCC generally had more advanced TNM-stage and grade, and were significantly more likely than type 1 pRCC to have metastasis at diagnosis (30.0% for type 2 vs. 8.7% for type 1). This is a markedly higher proportion than reported by Pignot et al. (16.1% for type 2 vs. 4.4% for type 1) in a cohort of 130 pRCC patients [33]. However, their study only included patients that underwent surgery, while 11.2% of our cohort were notoperated on. Although, type 2 pRCC more often diagnosed on stage IV than type 1 pRCC tumors, when grades 1 and 2 were combined and compared to grades 3 and 4, there was a significant difference between the two pRCC subtypes, which is in line with the findings of other studies [33, 34].

### Strenghts and limitations

The main strength of this study is the whole nation coverage of consecutive RCC cases. Similarly, we included both surgical and non-surgical patients. Another strength is that our access to centralized follow-up data allowed for robust OS and CSS estimations. Limitations include the retrospective nature of the study and small number of patients (*n* = 143) with pRCC subtypes. Finally, even though all 158 patients were included in our incidence and mortality analysis, 15 patients could not be subtyped and were therefore excluded from subtype comparisons.

## Conclusions

pRCC constitutes a somewhat smaller proportion of RCCs in Iceland than described in other studies. The incidence of pRCC has been rising over the last five decades, especially for males, which is mainly driven by an increase in the type 1 pRCC subtype. However, with no change in pRCC mortality and an increasing incidence, survival appears to be improving. Finally, although type 1 pRCC patients were diagnosed at lower grades and TNM stages than type 2 pRCC patients, pRCC subtyping did not predict disparity in outcome after multivariable adjustment. Our results are therefore in line with the 5^th^ Edition of WHO classification of Urinary and Male Genital Tumours, supporting that TNM stage and advanced nuclear grade seem to be more relevant for estimating the outcome of patients with pRCC.

### Supplementary Information

Supplementary Material 1: Figure S1. Estimated CSS for pRCC patients diagnosed 1971-2020 with pRCC on different TNM stages. Figure S1 shows estimated CSS depending on TNM stage for pRCC-1 and 2 taken together. One-year CSS for TNM stage I-IV was 98.7% (95% CI: 0.98, 1), 95.1% (95% CI: 0.92, 0.99), 88.8% (95% CI: 0.86, 0.92) and 42.9% (95% CI: 0.37, 0.49), respectively, and 5-year CSS was 96.5% (95% CI: 0.95, 98), 82.8% (95% CI: 0.77, 0.89), 72.4% (95% CI: 0.68, 0.78) and 14.4% (95% CI: 0.11, 0.19) for the same stages. Five-year OS was 85% (95% CI: 0.82, 0.88), 72.6% (95% CI: 0.66, 0.80), 62.5% (95% CI: 0.58, 0.68) and 12.6% (95% CI: 0.09, 0.17), for TNM stages I-IV, respectively.

Supplementary Material 2: Figure S2. Estimated CSS for pRCC patients diagnosed 1971-2020 with pRCC on different Fuhrman grades. Survival for different pRCC Fuhrman-grade groups is shown in Figure S2. Five-year CSS for grade 1 and 2 combined was 88.9% (CI: 0.82, 0.96) and 66.0% for grade 3 and 4 (CI: 0.53, 0.82).

## Data Availability

The data underlying this article will be shared on reasonable request to the corresponding author.
