# Supplementary figures and images for: An epidemiological and clinicopathological study of type 1 vs. type 2 morphological subtypes of papillary renal cell carcinoma– results from a nation-wide study covering 50 years in Iceland
Source: BMC Urol. 2024 May 13;24:105. doi: 10.1186/s12894-024-01494-9 (PMC11089793; doi:10.1186/s12894-024-01494-9)

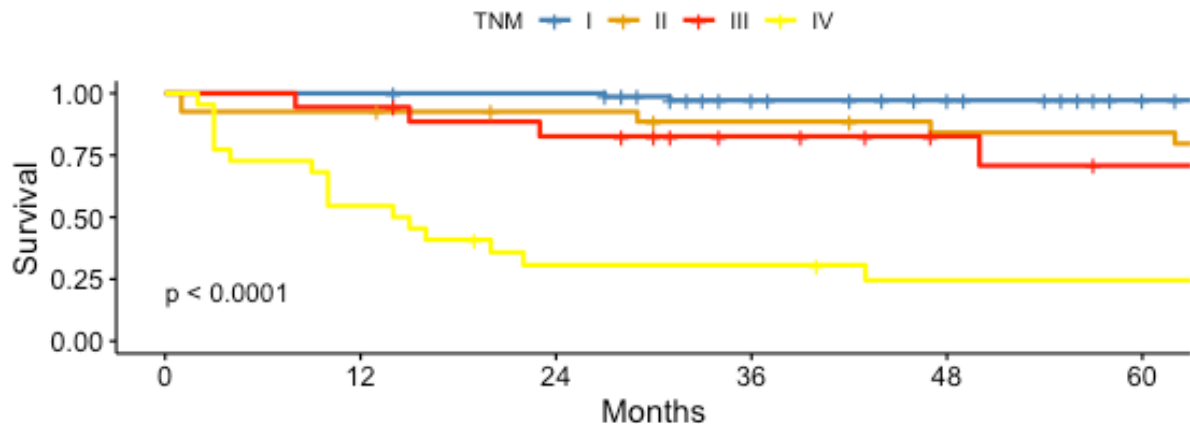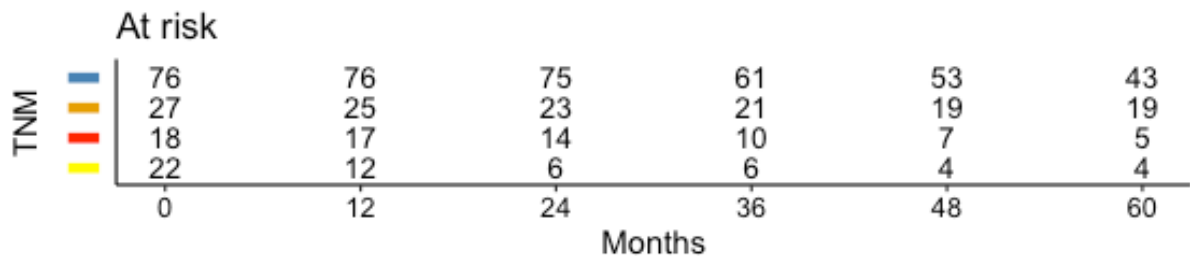

Supplement: Supplementary file 1 — Supplementary Material 1: Figure S1. Estimated CSS for pRCC patients diagnosed 1971-2020 with pRCC on different TNM stages. Figure S1 shows estimated CSS depending on TNM stage for pRCC-1 and 2 taken together. One-year CSS for TNM stage I-IV was 98.7% (95% CI: 0.98, 1), 95.1% (95% CI: 0.92, 0.99), 88.8% (95% CI: 0.86, 0.92) and 42.9% (95% CI: 0.37, 0.49), respectively, and 5-year CSS was 96.5% (95% CI: 0.95, 98), 82.8% (95% CI: 0.77, 0.89), 72.4% (95% CI: 0.68, 0.78) and 14.4% (95% CI: 0.11, 0.19) for the same stages. Five-year OS was 85% (95% CI: 0.82, 0.88), 72.6% (95% CI: 0.66, 0.80), 62.5% (95% CI: 0.58, 0.68) and 12.6% (95% CI: 0.09, 0.17), for TNM stages I-IV, respectively. [file 12894_2024_1494_MOESM1_ESM.pdf]

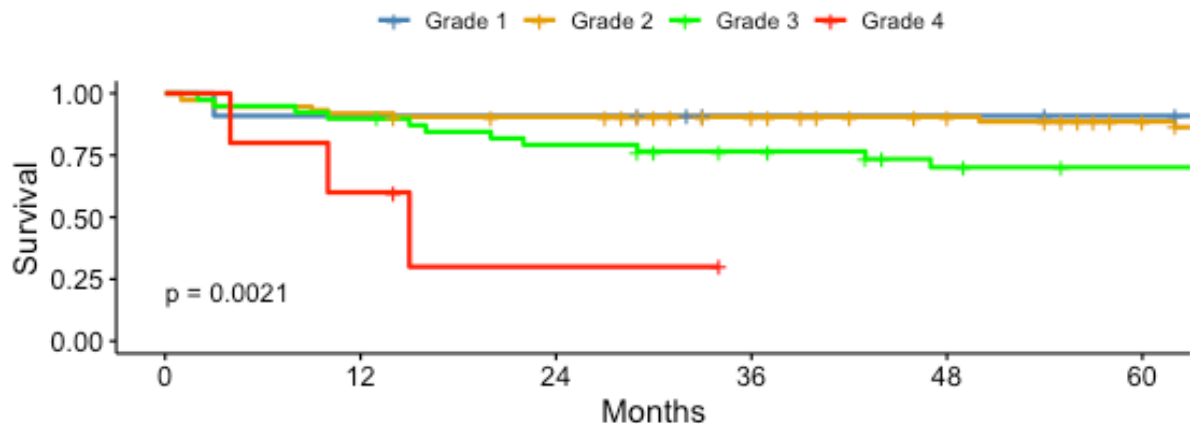

At risk

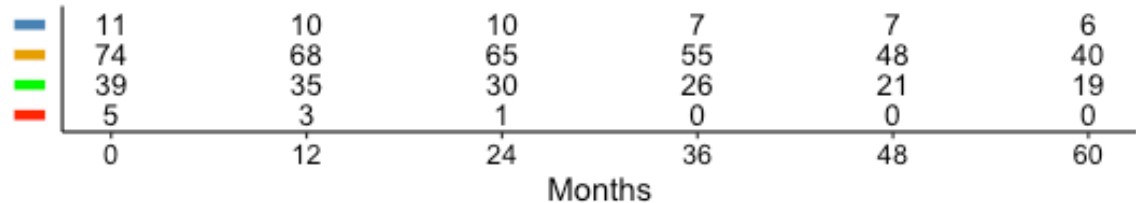

Supplement: Supplementary file 2 — Supplementary Material 2: Figure S2. Estimated CSS for pRCC patients diagnosed 1971-2020 with pRCC on different Fuhrman grades. Survival for different pRCC Fuhrman-grade groups is shown in Figure S2. Five-year CSS for grade 1 and 2 combined was 88.9% (CI: 0.82, 0.96) and 66.0% for grade 3 and 4 (CI: 0.53, 0.82). [file 12894_2024_1494_MOESM2_ESM.pdf]
